# Editorial for the Special Issue on Glassy Materials Based Microdevices

**DOI:** 10.3390/mi10010039

**Published:** 2019-01-08

**Authors:** Giancarlo C. Righini, Nicoletta Righini

**Affiliations:** 1 “Enrico Fermi” Historical Museum of Physics and Study & Research Centre, 00184 Roma, Italy; 2 “Nello Carrara” Institute of Applied Physics (IFAC), CNR. 50019 Sesto Fiorentino, Italy; 3 Research Institute on Ecosystems and Sustainability (IIES), National Autonomous University of Mexico (UNAM), 58190 Morelia, Mexico

Glassy materials, i.e., glasses and most polymers, play a very important role in microtechnologies and photonics. Both are substances frozen in a liquid-like structure and are characterized by a glass transition temperature. The excellent properties of glasses have made them fundamental for the development of optical and photonic components, from very large sizes (e.g., telescope lenses) down to the micrometric scale (e.g., microlenses and microresonators). Polymers, too, generally do not crystalize but form amorphous solids: their glassy properties are important for many applications such as nanolithography. This special issue of *Micromachines,* entitled “Glassy Materials Based Microdevices”, contains 19 papers (five reviews and 14 research articles) which highlight recent advances in microdevices and microtechnologies exploiting the properties of glassy materials. Contributions were solicited from both leading researchers and emerging investigators.

Several of these papers deal with the fabrication, physics and applications of glass and polymer microspheres, which constitute a very simple but very intriguing type of microdevice. A broad overview of the smart uses of solid and hollow glass microspheres in the field of energy, from solar cells to hydrogen storage and nuclear fusion, is presented in the review paper by Righini [1]. Polystyrene microspheres are used in the article by Piccolo et al. to construct a two-dimensional grating for the development of a low-cost chromatic sensor able to simultaneously determine the vectorial strain–stress information in the x and y directions [2]. As it is well known, microspheres may also operate as resonating cavities, where the light is trapped at the surface in whispering gallery modes (WGM). Yu et al. review the fabrication methods of microspherical resonators using various compound glasses, including heavy metal oxide glasses and chalcogenide glasses, and present some applications, e.g., lasing and sensing [3]. The critical issue of the robust coupling of light into a glass microspherical resonator is discussed in the article by Chiavaioli et al., who present a comprehensive model for designing an all-in-fiber sensing set-up and validate it by comparing the simulated results with the experimental ones [4]. Another aspect of light coupling in and out a WGM resonator is examined in the article by Konstantinou et al., where the implementation of a three-port, light guiding and routing T-shaped configuration based on the combination of a WGM microresonator and micro-structured optical fibers is demonstrated [5]. To complete this group of papers, the work by Li et al. illustrates the potential of an optofluidic hollow microsphere (microbubble) resonator for the highly sensitive label-free detection of small molecules and drug screening [6].

Microfluidics is fundamental for the development of biomedical sensing and analysis microsystems. Glass has proved to be a very convenient substrate for microfluidic chips thanks to its insulating properties, mechanical resistance and high solvent compatibility. The prototyping of microfluidic devices in low quantities may be time-consuming and expensive; the article by Wlodarczyk et al. describes a laser-based process that enables the fabrication of a fully-functional microfluidic device in less than two hours by using two thin glass plates [7]. The femtosecond laser irradiation followed by chemical etching (FLICE) technique was used by Italia et al. to fabricate a buried microfluidic device in a silica substrate; the design was optimized to minimize the diffusive mass transfer between two laminar flows [8]. The fabrication of glass microfluidic chips by a molding process that requires only tens of minutes and therefore appears to be a promising method for fast prototyping and mass production of microfluidic chips is described in the article by Wang et al. [9]. A microfluidic flow cytometer fabricated in polydimethylsiloxane (PDMS) by using a SU-8 photoresist mold was employed by Fan et al. for single cell analysis; data about the expression of β-actin proteins in ~10,000 single cells were obtained [10].

Precision glass molding and micropatterning technologies are of paramount importance in other fields, too. Zhou et al. provide a review of the fabrication technique of infrared aspherical lenses and microstructures in chalcogenide glass through precision glass molding [11]. Micropatterning of metal substrates, in particular the forming of microdomes on an aluminum substrate, is described in the article by Kim et al. [12]. The silicon–glass platform is at the base of the MEMS technology, which has been exploited by Knapkiewicz for the construction of high-vacuum self-pumping cells that are fully suitable for atomic spectroscopy [13]. The scribing of glass for subsequent dicing may be critical in the manufacturing of some microcircuits and microdevices; Zhang et al. discuss and experimentally test a method involving micro-crack-induced severing in order to realize the rapid and precision cleaving of the hard quartz glass in chip materials [14].

Many microdevices find application in the field of photonics. The paper by Amiri et al. provides a basic introduction to optical waveguides and to their applications, with special attention to fiber Bragg gratings for sensing applications [15]. Materials are very important both in microelectronics and in photonics: Falcony et al. provide an overview of the spray pyrolysis technique, with the focus on the research work performed in relation to the synthesis of high-K dielectric and luminescent materials in the form of coatings and powders as well as multiple layered structures [16]. The sol-gel technique is also extensively used to synthesize optical materials, especially glassy materials doped with rare earths; in the article by Trejo-García et al., the synthesis and spectroscopic characterization of Eu^3+^-doped hybrid silica–poly(methyl methacrylate) (PMMA) material is presented [17]. The photoluminescent properties of rare earth-doped glasses are discussed in the article by Enrichi et al., with reference to the broadband sensitization effect of Yb^3+^ ions due to the energy transfer from silver dimers/multimers [18]. Frequency conversion processes, based on efficient light excitation and re-emission in rare earth ions, may be exploited to increase the performance of silicon solar cells, and the article by Quandt et al. discusses the modelling of up-conversion processes, in particular, in the context of solar cell device simulations, showing their potential for the proper design of new types of highly efficient solar cells [19].

We would like to thank all the authors for their submissions to this special issue. We also thank all the reviewers for dedicating their time and helping ensure the quality of the submitted papers, and, last but not least, the staff at the editorial office of *Micromachines* for their efficient assistance.
